# Dietary *Lactobacillus rhamnosus* GG Supplementation Improves the Mucosal Barrier Function in the Intestine of Weaned Piglets Challenged by Porcine Rotavirus

**DOI:** 10.1371/journal.pone.0146312

**Published:** 2016-01-04

**Authors:** Xiangbing Mao, Changsong Gu, Haiyan Hu, Jun Tang, Daiwen Chen, Bing Yu, Jun He, Jie Yu, Junqiu Luo, Gang Tian

**Affiliations:** 1 Animal Nutrition Institute, Sichuan Agricultural University, Yucheng District, Ya’an, People’s Republic of China; 2 Key Laboratory of Animal Disease-Resistance Nutrition, Ministry of Education, China, Ya’an, People’s Republic of China; Indian Institute of Science, INDIA

## Abstract

*Lactobacillus rhamnosus* GG (LGG) has been regarded as a safe probiotic strain. The aim of this study was to investigate whether dietary LGG supplementation could alleviate diarrhea via improving jejunal mucosal barrier function in the weaned piglets challenged by RV, and further analyze the potential roles for apoptosis of jejunal mucosal cells and intestinal microbiota. A total of 24 crossbred barrows weaned at 21 d of age were assigned randomly to 1 of 2 diets: the basal diet and LGG supplementing diet. On day 11, all pigs were orally infused RV or the sterile essential medium. RV infusion increased the diarrhea rate, increased the RV-Ab, NSP4 and IL-2 concentrations and the Bax mRNA levels of jejunal mucosa (*P*<0.05), decreased the villus height, villus height: crypt depth, the sIgA, IL-4 and mucin 1 concentrations and the ZO-1, occludin and Bcl-2 mRNA levels of jejunal mucosa (*P*<0.05), and affected the microbiota of ileum and cecum (*P*<0.05) in the weaned pigs. Dietary LGG supplementation increased the villus height and villus height: crypt depth, the sIgA, IL-4, mucin 1 and mucin 2 concentrations, and the ZO-1, occludin and Bcl-2 mRNA levels of the jejunal mucosa (*P*<0.05) reduced the Bax mRNA levels of the jejunal mucosa (*P*<0.05) in weaned pigs. Furthermore, dietary LGG supplementation alleviated the increase of diarrhea rate in the weaned pigs challenged by RV (*P*<0.05), and relieve the effect of RV infection on the villus height, crypt depth and the villus height: crypt depth of the jejunal mucosa (*P*<0.05), the NSP4, sIgA, IL-2, IL-4, mucin 1 and mucin 2 concentrations of jejunal mucosa (*P*<0.05), the ZO-1, occludin, Bax and Bcl-2 mRNA levels of the jejunal mucosa (*P*<0.05), and the microbiota of ileum and cecum (*P*<0.05) in the weaned pigs challenged by RV. These results suggest that supplementing LGG in diets alleviated the diarrhea of weaned piglets challenged by RV via inhibiting the virus multiplication and improving the jejunal mucosal barrier function, which was possibly due to the decreasing apoptosis of jejunal mucosal cells and the improvement of intestinal microbiota.

## Introduction

Lactobacilli are the normal inhabitants in the gut of humans and animals. With Lactobacilli having a positive role for regulating the intestinal microbiota, many studies during the past decades have shown that Lactobacilli have the specific probiotic properties, including maintaining the gastrointestinal tract health [1–4]. *Lactobacillus rhamnosus* GG (LGG), isolated by Glodin and Gorbach from the healthy adult’s faece [5–6], has been shown some probiotic characteristics, including high adhesion capacity *in vitro*, high antimicrobial activity against pathogens by some substances, and high resistance against gastric acidity [7–9]. Therefore, LGG has been regarded as a safe probiotic strain.

Rotavirus (RV) is a double-stranded RNA icosahedral RNA virus, which is a kind of the major pathogen that can induce severe gastrointestinal damage and diarrhea in young animals and children [10, 11]. The previous studies have also shown that RV mainly damages the mucosal barrier of the proximal small intestine [12], which could be due that it can induce oxidative stress that could further lead to apoptosis in the intestinal epithelial cells [13–15].

Recent studies have shown that oral administration of LGG could prevent and attenuate some types of diarrhea [16, 17]. In some studies and clinical trials, LGG therapy has also decreased RV diarrhea of animals and children [15, 18–21]. Additionally, this could be due that LGG treatment relieves the effect of RV on the barrier function in the intestine of gnotobiotic piglets, which is relative with improving microbiota, and decreasing autophagy and increasing apoptosis in the ileal epithelial cells [21–24]. However, there are some differences between physiological function of gnotobiotic animals and that of normal ones [25, 26]. Thus, the LGG treatment regulating intestinal barrier function and the related mechanism in normal animals and children could be different with that in gnotobiotic ones.

Our recent study has also shown that dietary LGG supplementation could alleviate the effect of RV challenge on the intestinal antioxidant capacity of weaning piglets [15]. The current study was conducted to test the hypothesis that dietary LGG supplementation could alleviate the diarrhea via improving the jejunal mucosal barrier function in weaned piglets challenged by RV. In addition, during this process, the potential roles for apoptosis of jejunal mucosal cells and intestinal microbiota would be analyzed.

## Materials and Methods

### Bacterial strain, growth and storage conditions

*Lactobacillus rhamnosus* GG, a generous gift from Professor Shiyan Qiao (China Agricultural University, China), was anaerobically propagated on sterile Man Rogosa Sharpe medium at 37°C for 24 h, and the culture was centrifuged for 10 min at 5000 × g and 4°C. Then, the cells were resuspended in reconstituted skim milk (20% w/v), which was immediately freeze-dried. The freeze-dried powder containing 1.5 × 10^10^ colony forming units (CFU)/g was stored in the sealed packet at 4°C until used.

### Animals and diets

The Animal Care Advisory Committee of Sichuan Agricultural University approved the experimental protocol. A total of 24 crossbred (Duroc × Large White × Landrace) barrows, weaned at 21 d of age, were individually housed in the metabolism cage (1.5 × 0.7 × 1.0 m^3^). The room lighting was natural, and the room temperature was maintained at 25–28°C. The diets were supplied 4 times daily at 0800, 1200, 1600 and 2000 h, and water could be freely accessed for the piglets. During the experiment, the health of all pigs was monitored for 4 times every day before the piglets were fed, and there were not any unexpected illness or deaths.

A corn- and soybean meal-based diet was formulated to approximately meet National Research Council-recommended nutrient requirements for pigs weighing 5–10 kg (NRC 2012) [27], which was shown in Table 1. The LGG supplementing diet was the basal diet supplemented with 10^9^ CFU/g LGG.

**Table 1:** The composition and nutrient content of basal diets.

Items	Content
Ingredient composition, %	
Corn	29.585
Extruded corn	29.585
Extruded soybean	12.00
Soybean meal	11.00
Soybean protein concentrate	5.50
Fish meal	5.00
Whey powder	3.00
Soybean oil	2.00
L-Lysine-HCl	0.22
L-Threonine	0.06
DL-Methionine	0.12
Choline chloride	0.15
NaCl	0.30
CaCO_3_	0.40
CaHPO_4_	0.75
Premix^1^	0.33
Total	100.00
Nutrient levels^2^, %	
Digestible energy, Mcal/kg	3.547
Crude protein	20.93
Total lysine	1.33
Total methionine and cystine	0.71
Total threonine	0.74
Total tryptophan	0.21
Calcium	0.80
Phosphorus available	0.44

^1^ Provided the folloing per kg of diet: Vitamin A, 9000 IU; Vitamin D_3_, 3000 IU; Vitamin E, 20.0 IU; Vitamin K_3_, 3.0 mg; Vitamin B_1_, 1.5 mg; Vitamin B_2_, 4.0 mg; Vitamin B_6_, 3.0 mg; Vitamin B_12_, 0.2 mg; Nicotonic, 30.0 mg; Pantothenic, 15.0 mg; Folic acid, 0.75 mg; Biotin, 0.1 mg; Fe (as FeSO_4_·7H_2_O), 96.0 mg; Cu (as CuSO_4_·5H_2_O) 84.0 mg; Zn (as ZnSO_4_·7H_2_O), 93 mg; Mn (as MnSO_4_·H_2_O), 4.0 mg; I (as KI) 0.14 mg; Se (as Na_2_SeO_3_·5H_2_O) 0.30mg.

^2^ Calculated nutrient levels.

### Experimental design and sample collection

After 3 d of acclimatization, based on the initial body weights and origin of litters, twenty four crossbred barrows were weighed (6.69 ± 0.32 kg) and allotted randomly to one of two dietary treatments (n = 12): 1) the corn- and soybean meal-based diet as control; 2) the LGG supplementing diet as the LGG treatment. On day 15, all piglets were orally infused with 5 mL of the sterile 100 mM sodium bicarbonate solution. Then, half of the piglets on each diet were orally infused with 3 mL (1.4 × 10^7^ Tissue culture infective dose 50 (TCID_50_)/mL) of porcine rotavirus (PRV) dissolved in the essential medium, while the other half were orally administrated with the same amount of the sterile essential medium. Following PRV administration, the diarrhea of all pigs was observed. Fecal consistency was scored as follows: 0, normal; 1, pasty; 2, semiliquid; and 3, liquid. Pigs with daily fecal consistency scores of ≥ 2 were considered diarrheic [28].

On d 20, following weighing, all piglets were killed by an intracardial injection of Na pentobarbital (50 mg/kg body weight) and jugular exsanguinations. Following the small intestine was removed, a 25-cm tissue section was rapidly excised at 50% of the length of the small intestine, rinsed with ice-cold physiological saline, and blotted up on paper. The 3-cm segment of the jejunum was fixed in 10% neutral-buffered formalin for the analysis of histomorphology. The mucosa from this residual jejunum section was sequentially obtained through carefully scraping of the mucosal layer with a glass microscope slide, immediately frozen in liquid nitrogen, and stored at -80°C for real-time quantitative PCR, ELISA and Western Blot analysis. Approximately 3 g of the digesta from the ileum and cecum were kept in sterile tubes and immediately frozen at -80°C for microbial DNA analysis.

### Porcine rotavirus preparation and virus titre determination

PRV in present study was a tissue culture-adapted Ohio State University (OSU) strain (ATCC #VR-893), which was propagated in the MA104 cell that was obtained from China Center for Type Culture Collection as described previously [29]. Briefly, the PRV activated by 5 μg/mL trypsin (type IX, Sigma) for 30 min at 37°C was inoculated with the MA104. Following 2 h of incubation at 37°C, MA104 cells were washed three times with sterile PBS, and then incubated at 37°C in Eagle minimal essential medium (MEM). When the extensive cytopathic effect was observed with microscope, the culture was frozen and thawed three times, and centrifuged at 3000 × g for 10 min. The supernatant containing the PRV was stored at -80°C.

The virus titre (TCID_50_ value) was measured as described previously [30]. Briefly, the MA104 cell was grown to 80–90% confluence in ninety-six-well plates, and then infected with 50 μL aliquots of 1:10 serial dilutions (in the MEM medium) of PRV samples (8 wells/dilution). After the incubation for 4 d at 37°C in 5% CO_2_, the cytopathic effect was visualized through staining the remaining viable cells with crystal violet. The virus titre was calculated with the Speaman method (Speaman 1908) and expressed as log_10_ (TCID_50_) [31].

### Analysis of the histomorphology in the jejunum

The jejunal histomorphology was determined as described previously [30]. Briefly, following the fixing, the segment of the jejunum was embedded in paraffin. Then, consecutive sections (5 μm) were stained with hematoxylin-eosin. The villus height and crypt depth of the jejunal mucosa were measured at 40 × magnification with an Olympus CK 40 microscope (Olympus Optical Company).

### Analysis of rotavirus antibody (RV-Ab), rotavirus non-structural protein 4 (NSP4), sIgA, IL-2, IL-4, and mucin1 and 2 concentrations in the jejunal mucosa

The RV-Ab concentrations of the jejunal mucosa in pigs and the NSP4 of rotavirus were determined using the commercially available enzyme-linked immunosorbent assay (ELISA) kits from Nuoyuan Co., Ltd. (Shanghai, China). The sIgA, IL-2 and IL-4 concentrations of the jejunal mucosa in pigs were determined using the commercially available enzyme-linked immunosorbent assay (ELISA) kits from TSZ ELISA (Framingham, MA) according to the manufacturer’s instructions. The mucin 1 and mucin 2 of the jejunal mucosa in pigs were measured by using the commercially available mucin 1 and 2 ELISA kits from CUSABIO Biotech Co. Ltd. (Wuhan, China) according to the manufacturer’s instructions. The concentrations of RV-Ab, NSP4, sIgA, IL-2, IL-4 mucin 1 and mucin 2 were quantified by using a BioTek Synergy HT microplate reader (BioTek Instruments, Winooski, VT), and absorbance was measured at 450 nm.

### Real-time quantitative PCR for zonula occludens 1 (ZO-1), occludin, B-cell lymphoma/leukaemia-2 (Bcl-2) and B-cell lymphoma/leukaemia-2-associated X protein (Bax)

Total RNA was isolated from the jejunal mucosa by using TRIZOL reagent (TaKaRa Biotechnology (Dalian) Co., Ltd., Dalian, China) according to the manufacturer’s instructions. The RNA quality was determined by DU 640 UV spectrophotometer detection (Beckman Coulter Inc., Fullerton, CA), and the OD_260_:OD_280_ ratio ranged from 1.8 and 2.0 in all samples. The RNA integrity was analyzed by 1% agarose gel electrophoresis. The RNA samples were reversely transcribed into complementary DNA by using RT Reagents (TaKaRa Biotechnology (Dalian) Co., Ltd., Dalian, China) according to the manufacturer’s instruction. Following reverse transcription, expression levels of ZO-1, occludin, Bcl-2, Bax and β-actin in the jejunal mucosa were analyzed by real-time quantitative PCR using SYBR Premix Ex Taq reagents (TaKaRa Biotechnology (Dalian) Co., Ltd., Dalian, China) and CFX-96 Real-Time PCR Detection System (Bio-Rad Laboratories, Richmond, CA) as previously described [32]. The primers were purchased by TaKaRa Biotechnology (Dalian) Co., Ltd. (Dalian, China), which was listed in Table 2. Relative gene expression to the reference gene (β-actin) was determined in order to correct for the variance in amounts of RNA input in the reaction. In addition, the relative gene expressions compared to the reference gene were calculated with the previous method [33].

**Table 2:** Primer sequences used for real-time quantitative PCR.

Gene	Nucleotide sequences 5ˊ-3ˊ	Annealing temperature (°C)	Product size (bp)
ZO-1	Forward: TGGCATTATTCGCCTTCATAC	59.0	171
	Reverse: AGCCTCATTCGCATTGTTT		
occludin	Forward: CTACTCGTCCAACGGGAAAG	61.5	158
	Reverse: ACGCCTCCAAGTTACCACTG		
Bcl-2	Forward: TGCCTTTGTGGAGCTGTATG	60.0	144
	Reverse: GCCCGTGGACTTCACTTATG		
Bax	Forward: AAGCGCATTGGAGATGAACT	60.0	121
	Reverse: TGCCGTCAGCAAACATTTC		
β-actin	Forward: TCTGGCACCACACCTTCT	61.5	114
	Reverse: TGATCTGGGTCATCTTCTCAC		

### Bacterial DNA isolation and microbial real-time quantitative PCR

Bacterial DNA in the ileal and cecal digesta was extracted by using the Stool DNA Kit (Omega Bio-tek) according to the manufacturer’s instruction. The microbial real-time quantitative PCR was determined as described previously [34]. Briefly, the number of total bacteria was analyzed by real-time quantitative PCR using SYBR Premix Ex Taq reagents (TaKaRa Biotechnology (Dalian) Co., Ltd., Dalian, China) and CFX-96 Real-Time PCR Detection System (Bio-Rad Laboratories, Richmond, CA), and the number of *Lactobacillus*, *E*. *coli* and *Bifidobacterium* was analyzed by real-time quantitative PCR using PrimerScriptTM PCR kit (Perfect Real Time; TaKaRa Biotechnology (Dalian) Co., Ltd., Dalian, China) and CFX-96 Real-Time PCR Detection System (Bio-Rad Laboratories, Richmond, CA) as previously described (Chen et al., 2013). All primers and probes were purchased by TaKaRa Biotechnology (Dalian) Co., Ltd. (Dalian, China), which was listed in Table 3. For the quantification of bacteria in the test samples, specific standard curves were generated by constructing standard plasmids as presented by Chen et al. (2013) [34]. In addition, bacterial copies were transformed (log_10_) before statistical analysis.

**Table 3:** Primer and probe sequences used for real-time quantitative PCR.

Bacteria	Nucleotide sequences (5'-3')	Annealing temperature (°C)	Product size (bp)
*Bifidobacterium*	Forward: CGCGTCCGGTGTGAAAG	55.0	121
	Reverse: CTTCCCGATATCTACACATTCCA		
	Probe: ATTCCACCGTTACACCGGGAA		
*Lactobacillus*	Forward: GAGGCAGCAGTAGGGAATCTTC	53.0	126
	Reverse: CAACAGTTACTCTGACACCCGTTCTTC		
	Probe: AAGAAGGGTTTCGGCTCGTAAAACTCTGTT		
*Escherichia coli*	Forward: CATGCCGCGTGTATGAAGAA	55.0	96
	Reverse: CGGGTAACGTCAATGAGCAAA		
	Probe: AGGTATTAACTTTACTCCCTTCCTC		
Total bacteria	Forward:ACTCCTACGGGAGGCAGCAG	61.5	200
	Reverse:ATTACCGCGGCTGCTGG		

### Statistical analysis

All data were analyzed as a 2 × 2 factorial with the general linear model procedures of the SAS (Version 8.1; SAS Institute, Gary, NC). The factors of models included the main effects of LGG treatment (supplemented or unsupplemented with LGG in the diet) and PRV challenge (PRV or sterile essential medium) as well as their interaction. *P*<0.05 was considered to indicate statistical significance, and *P*<0.10 was considered to indicate statistical tendency. All data were expressed as mean ± standard error.

## Results

### The effect of dietary LGG supplementation and/or RV infusion on the diarrhea, and the RV-Ab and NSP4 concentrations of jejunal mucosa in the weaned pigs

After RV infusion, the diarrhea rate and the RV-Ab and NSP4 concentrations of jejunal mucosa was increased in the weaned pigs (*P*<0.05; Tables 4 and 5). However, dietary LGG supplementation, to some extent, alleviated the increase of diarrhea rate and NSP4 concentration of jejunal mucosa (*P*<0.05), and further increased the RV-Ab concentration of jejunal mucosa (*P*<0.05) in the weaned pigs challenged by RV (Tables 4 and 5).

**Table 4:** The effect of dietary LGG supplementation and/or RV challenge on the diarrhea of weaned pigs (n = 6)^#^.

	RV -		RV +		*P*-value		
	CON	LGG	CON	LGG	LGG	RV	LGG×RV
Diarrhea rate (%)	0.00±0.00^C^	0.00±0.00^C^	68.00±10.20^A^	20.00±0.00^B^	<0.05	<0.05	<0.05

^#^ RV -, infusing the essential medium; RV +, infusing the porcine rotavirus; CON, basal diet; LGG, LGG-supplemented diet.

^A, B, C^ In the same row, values with different letter superscripts mean significant difference (*P*<0.05).

**Table 5:** The effect of dietary LGG supplementation and/or RV challenge on the contents of RV-Ab, NSP4, sIgA, IL-2, IL-4, mucin 1 and mucin 2 in the jejunal mucosa of weaned pigs (n = 6)^#^.

	RV -		RV +		*P*-value		
	CON	LGG	CON	LGG	LGG	RV	LGG×RV
RV-Ab (ng/mg protein)	5.54±0.32^C^	6.40±1.05^C^	19.30±1.59^B^	27.73±2.08^A^	<0.05	<0.05	<0.05
NSP4 (pg/mg protein)	55.82±4.39^C^	52.18±4.68^C^	187.01±11.35^A^	139.10±2.99^B^	<0.05	<0.05	<0.05
Mucin 1 (U/mg protein)	192.83±19.73^B^	351.62±36.68^A^	105.45±18.97^C^	162.77±18.25^B^	<0.05	<0.05	0.06
Mucin 2 (ng/mg protein)	13.81±1.26^B^^C^	24.95±2.53^A^	9.71±2.33^C^	19.44±3.20^A^^B^	<0.05	0.05	0.76
sIgA (μg/mg protein)	9.45±0.05^B^	10.46±0.34^A^	7.03±0.15^C^	9.37±0.27^B^	<0.05	<0.05	<0.05
IL-2 (pg/mg protein)	184.23±5.02^B^	190.59±10.09^B^	227.24±5.94^A^	199.87±7.73^B^	0.20	<0.05	<0.05
IL-4 (pg/mg protein)	24.29±0.92^B^	28.43±0.59^A^	19.40±0.41^C^	23.58±0.82^B^	<0.05	<0.05	0.99

^#^ RV -, infusing the essential medium; RV +, infusing the porcine rotavirus; CON, basal diet; LGG, LGG-supplemented diet.

^A, B, C^ In the same row, values with different letter superscripts mean significant difference (*P*<0.05).

### The effect of dietary LGG supplementation and/or RV infusion on the concentrations of sIgA, IL-2, IL-4, mucin 1 and mucin 2 in the jejunal mucosa of weaned pigs

RV infusion decreased the concentrations of sIgA (*P*<0.05), IL-4 (*P*<0.05), mucin 1 (*P*<0.05) and mucin 2 (*P* = 0.05), and increased the IL-2 levels (*P*<0.05) in the jejunal mucosa of weaned pigs (Table 5). However, dietary LGG supplementation increased the sIgA, IL-4, mucin 1 and mucin 2 concentrations of the jejunal mucosa in weaned pigs (*P*<0.05; Table 5). Furthermore, in the weaned pigs challenged by RV, the effect of RV infusion on the sIgA, IL-2, IL-4, mucin 1 and mucin 2 concentrations of the jejunal mucosa could be alleviated by dietary LGG supplementation (*P*<0.05).

### The effect of dietary LGG supplementation and/or RV infusion on the morphology of the jejunal mucosa in the weaned pigs

The morphology of the jejunal mucosa in the weaned pigs was shown in the S1 Fig. Following PRV infusion, the villus height and villus height: crypt depth were decreased in the weaned pigs (*P*<0.05; Table 6). However, supplementing LGG in the diet increased the villus height and villus height: crypt depth (*P*<0.05), and tended to reduce the crypt depth (*P* = 0.08) of the jejual mucosa in the weaned pigs (Table 6). Moreover, in the weaned pigs challenged by RV, dietary LGG supplementation could improve the effect of RV infusion on the villus height, crypt depth and the villus height: crypt depth of the jejunal mucosa (*P*<0.05; Table 6).

**Table 6:** The effect of dietary LGG supplementation and/or RV challenge on the jejunal morphology of weaned pigs (n = 6)^#^.

	RV -		RV +		*P*-value		
	CON	LGG	CON	LGG	LGG	RV	LGG×RV
Villus height (μm)	262.61±3.57^A^^B^	281.33±8.50^A^	202.70±9.56^C^	251.83±8.94^B^	<0.05	<0.05	0.08
Crypt depth (μm)	105.06±2.35^B^	106.76±2.74^B^	120.96±6.82^A^	102.62±4.32^B^	0.08	0.20	<0.05
Villus height:crypt depth	2.58±0.04^A^^B^	2.69±0.06^A^	1.70±0.04^C^	2.53±0.02^B^	<0.05	<0.05	<0.05

^#^ RV -, infusing the essential medium; RV +, infusing the porcine rotavirus; CON, basal diet; LGG, LGG-supplemented diet.

^A, B, C^ In the same row, values with different letter superscripts mean significant difference (*P*<0.05).

### The effect of dietary LGG supplementation and/or RV infusion on the gene expression of tight junction and apoptosis in the jejunal mucosa of weaned pigs

RV infusion decreased the mRNA levels of ZO-1, occludin and Bcl-2 (*P*<0.05), and increased the Bax mRNA levels (*P*<0.05) in the jejunal mucosa of weaned pigs (Table 7). However, dietary LGG supplementation increased the mRNA levels of ZO-1, occludin and Bcl-2 (*P*<0.05), and reduced the Bax mRNA levels (*P*<0.05) in the jejunal mucosa of weaned pigs (Table 7). Furthermore, in the weaned pigs challenged by RV, the effect of RV infusion on the ZO-1, occludin, Bax and Bcl-2 mRNA levels of the jejunal mucosa could be alleviated by dietary LGG supplementation (*P*<0.05; Table 7).

**Table 7:** The effect of dietary LGG supplementation and/or RV challenge on the gene expression of tight junction and apoptosis in the jejunal mucosa of weaned pigs (n = 6)^#^.

	RV -		RV +		*P*-value		
	CON	LGG	CON	LGG	LGG	RV	LGG×RV
ZO-1	1.00±0.12^C^	3.53±0.52^A^	0.28±0.06^C^	2.11±0.24^B^	<0.05	<0.05	0.28
occludin	1.00±0.11^B^	1.96±0.13^A^	0.57±0.19^B^	1.65±0.14^A^	<0.05	<0.05	0.67
Bax	1.00±0.08^B^	0.65±0.10^B^	1.76±0.28^A^	0.54±0.03^B^	<0.05	0.06	<0.05
Bcl-2	1.00±0.25^B^	2.62±0.56^A^	0.37±0.05^C^	1.39±0.14^B^	<0.05	<0.05	0.38

^#^ RV -, infusing the essential medium; RV +, infusing the porcine rotavirus; CON, basal diet; LGG, LGG-supplemented diet.

^A, B, C^ In the same row, values with different letter superscripts mean significant difference (*P*<0.05).

### The effect of dietary LGG supplementation and/or RV infusion on intestinal bacteria in the ileal and cecal digesta of weaned pigs

Following PRV infusion, *Lactobacillus* and total bacteria populations of ileum were decreased (*P*<0.05), *Lactobacillus* (*P* = 0.07) and *Bifidobacterium* (*P*<0.05) populations of cecum were reduced, and *E*. *coli* populations of ileum and cecum were increased (*P*<0.05) in the weaned pigs (Table 8). However, supplementing LGG in the diet increased *Lactobacillus* and total bacteria populations of ileum (*P*<0.05), enhanced *Lactobacillus* and *Bifidobacterium* populations of cecum (*P*<0.05), and reduce *E*. *coli* populations of ileum and cecum (*P*<0.05) in the weaned pigs (Table 8). What’s more, in the weaned pigs challenged by RV, dietary LGG supplementation could improve the effect of RV infusion on *Lactobacillus*, *Bifidobacterium* and *E*. *coli* populations of the ileum and/or cecum (*P*<0.05; Table 8).

**Table 8:** The effect of dietary LGG supplementation and/or RV challenge on intestinal bacteria in the ileal and cecal digesta of weaned pigs (log_10_ (copies/g)) (n = 6)^#^.

	RV -		RV +		*P*-value		
	CON	LGG	CON	LGG	LGG	RV	LGG×RV
Ileum							
*Lactobacillus*	4.32±0.03^C^	5.12±0.03^A^	3.69±0.04^D^	4.97±0.01^B^	<0.05	<0.05	<0.05
*Bifidobacterium*	2.41±0.12	2.28±0.64	2.04±0.17	2.00±0.28	0.83	0.38	0.91
*E*. *coli*	6.48±0.04^B^	5.72±0.03^D^	7.13±0.03^A^	6.36±0.02^C^	<0.05	<0.05	0.94
Total bacteria	10.38±0.04^B^	10.89±0.05^A^	9.73±0.07^C^	9.74±0.05^C^	<0.05	<0.05	<0.05
Cecum							
*Lactobacillus*	6.25±0.02^A^	6.27±0.03^A^	6.15±0.03^B^	6.27±0.01^A^	<0.05	0.07	0.11
*Bifidobacterium*	3.17±0.04^B^	4.14±0.05^A^	1.96±0.23^C^	3.53±0.04^B^	<0.05	<0.05	<0.05
*E*. *coli*	6.79±0.03^B^	6.27±0.03^C^	7.17±0.04^A^	7.11±0.03^A^	<0.05	<0.05	<0.05
Total bacteria	12.08±0.06	12.14±0.04	12.04±0.06	12.19±0.16	0.27	0.96	0.65

^#^ RV -, infusing the essential medium; RV +, infusing the porcine rotavirus; CON, basal diet; LGG, LGG-supplemented diet.

^A, B, C, D^ In the same row, values with different letter superscripts mean significant difference (*P*<0.05).

## Discussion

The important finding of the present work is that dietary LGG supplementation might alleviate the diarrhea of weaned piglets challenged by RV (Table 4), which could be due that supplementing LGG in the diet could reduce the virus multiplication and improve the jejunal mucosal barrier function, including non-specific barrier mechanisms and specific immunological responses (Tables 5–8).

RV is one of the main pathogens that may result in the serious diarrhea in the children and young animals [10, 11]. Our previous studies and some other studies have shown that RV infusion increased the diarrhea, and impaired the intestinal barrier functions in the pigs [15, 18–21], which was consistent with the current study (Tables 4–8). Moreover, this study reported that RV infusion increased the rotavirus antibody (RV-Ab) and rotavirus non-structural protein 4 (NSP4) levels in the jejunal mucosa of the weaned pigs (Table 5). These results indicated that the model of RV inducing intestinal barrier dysfunction was successful.

NSP4 of RV is an intracellular receptor that regulates the acquisition of a transient membrane envelope as subviral particles bud in the endoplasmic reticulum [35]. The further studies have shown that NSP4 is relative with the RV replication, and can act as a viral enterotoxin, which will induce the diarrhea in animals [36, 37]. In the current study, dietary LGG supplementation could alleviate the increasing NSP4 level of the jejunal mucosa that was induced by RV infusion in the weaned pigs (Table 5). This indicated that supplementing LGG in the diet might effectively inhibit the RV multiplication in the jejunal mucosa, which could be one of the factors that dietary LGG supplementation led to the decrease of diarrhea in pigs.

The non-specific barrier mechanisms in gut consist of the mucosal-epithelial integrity, intercellular junctions between the epithelial cells, and the mucus gel layer [38]. The intestinal mucosal morphology is the effective method of evaluating the surface area of the intestine undertaken for mucosal integrity [34, 38]. The intercellular junctions between the epithelial cells mainly formed from transmembrane proteins and nonmembrane proteins, including ZO-1 and occludin [39, 40]. In addition, mucins, such as mucin 1 and 2, are mainly secreted by gut goblet cells, which are the important components of the mucus gel layer in the intestinal mucosa [41]. The previous studies have shown that LGG administration can improve the intestinal mucosal morphology, and increase the expression of tight junction proteins and mucins [42–44]. Similar with these studies, this study also reported that dietary LGG supplementation could improve the mucosal morphology, the expression of ZO-1 and occluding, and the levels of mucin 1 and 2 in the jejunal mucosa of weaned pigs challenged by RV (Tables 5–7). These results indicated that LGG administration could improve the non-specific barrier mechanisms in the gut of pigs challenged by RV. However, Liu et al. (2013) has reported that human rotavirus (HRV) infusion may stimulate the expression of mucins and intercellular junction proteins, and gradually supplementing LGG in drinking water for 16 days decreases their expression in the intestinal epithelium of gnotobiotic pigs challenged by HRV [23]. The difference between our results and those of Liu et al. (2013) could be due whether the experimental pigs are gnotobiotic or not.

As the crucial component of specific immunological responses, sIgA plays an important role for protecting the intestinal mucosa against the pathogen invasion [45]. Recent *in vivo* and *in vitro* studies have shown that LGG treatment can improve the immunity, including the intestinal immunity [46–48]. Zhang et al. (2010) also reported that dietary LGG supplementation increased the sIgA levels in the jejunum and ileum of weaned pigs infused *E*. *coli* K88 [46]. Consistent with these studies, our current study showed that dietary LGG supplementation increased the sIgA levels in the jejunal mucosa of pigs, and could improve the effect of RV infusion on the sIgA levels of the jejunal mucosa (Table 5). Moreover, following the gut pathogen invasion, the intestinal-mucosal immune system can produce the specific antibody, which will benefit from the removal of pathogen. In this study, supplementing LGG in the diet might further enhance the RV-Ab in the jejunal mucosa of pigs challenged by RV (Table 5), which is similar with the previous study in the gnotobiotic pigs [49]. Therefore, LGG administration could improve the humoral immunity of intestinal mucosa in pigs challenged by different pathogens.

The T-helper (Th1/Th2) cytokine balance plays a crucial role in the immunity maintenance [50]. The previous studies have shown that LGG treatment affects the level of the T-helper (Th1/Th2) cytokine in mononuclear cells and monocyte-derived dendritic cells [51, 52], can decrease the IL-6 expression via upregulating the toll-like receptor 2 expression in porcine jejunal epithelial cell line (IPEC-J2) [47]. In addition, Kandasamy et al. (2014) has shown that LGG administration may regulate the IL-6 and IL-10 levels in the ileal mononuclear cells of gnotobiotic pigs challenged by HRV [53]. Similar with these studies, the current study also reported that dietary LGG supplementation could decrease the IL-2 level, and increase the IL-4 level in the jejunal mucosa of weaned pigs infused by RV (Table 5). These suggest that supplementing LGG in diets could improve the T-helper (Th1/Th2) cytokine imbalance of the jejunal mucosa induced by RV infusion, which would maintain the specific immunological responses in the intestine of weaned pigs. Furthermore, pro-inflammatory cytokines may increase intestinal permeability through the dysregulation of tight junction proteins [54, 55], which is the partial reason why LGG treatment could improve the ZO-1 and occludin mRNA levels in the jejunal mucosa of weaned pigs infused by RV (Table 7).

In our recent study, RV infusion induces the oxidative stress in the jejunal mucosa of pigs [15]. The oxidative stress plays a pivotal role in apoptosis [56]. This study also showed that RV infusion could increase the pro-apoptotic protein Bax mRNA level, and decrease the anti-apoptotic protein Bcl-2 mRNA level in the jejunal mucosa of weaned pigs (Table 7), which indicated that RV infusion increased the apoptosis of the jejunal mucosal cells. However, Lactobacilli, including LGG, have the antioxidant properties [57, 58]. Our recent study showed that dietary LGG supplementation could increase the antioxidant activity, and attenuate the oxidative stress in the intestinal mucosa of weaned pigs challenged by RV [15]. What’s more, in this study, supplementing LGG in the diet could increase the Bcl-2 mRNA level and decrease the Bax mRNA level in the jejunal mucosa of weaned pigs infused by RV (Table 7). These suggested that LGG administration could alleviate the increasing apoptosis of jejunal mucosal cells induced by RV challenge, which would improve the intestinal barrier integrity. However, Wu et al. (2013) got the opposite results in the experiment that utilizes the gnotobiotic pig as a research subject [21]. The difference between our results and those of Wu et al. (2013) could be due whether the experimental pigs are gnotobiotic or not.

Lactobacilli administration regulates the intestinal bacteria components of human and animal, including increasing the number of health-promoting bacterial species (such as Lactobacilli and Bifidobacteria) and decreasing the number of potential pathogenic bacterial species (such as *E*. *coli*) [59, 60], which is consistent with the results of the present study (Table 8). In addition, this study also showed that dietary LGG supplementation could improve the effect of RV infusion on the intestinal bacteria (Table 8), which is similar with the results in the gnotobiotic pigs [22]. The previous studies have reported that Bifidobacteria are the predominant populations in the large intestine [61], which could be the reason for the difference in Bifidobacteria species observed only in the cecum in this study. However, the intestinal microbiota is closely relative with the human and animal health via maintaining or improving the intestinal barrier function. Thus, dietary LGG supplementation improving the intestinal microbiota could also be an important reason that it could decrease the diarrhea of weaned pigs challenged by RV.

## Conclusion

RV infusion led to the diarrhea of weaned piglets via impairing the intestinal barrier function, but supplementing LGG in diets alleviated the diarrhea of weaned piglets challenged by RV via inhibiting the virus multiplication and improving the jejunal mucosal barrier function, which was possibly due to the decreasing apoptosis of jejunal mucosal cells and the improvement of intestinal microbiota.

## Supporting Information

S1 Fig The Jejunal mucosal morphology of the weaned pigs. (A) The pig fed the basal diet and orally infused with the sterile essential medium; (B) the pig fed the LGG supplementing diet and orally infused with the sterile essential medium; (C) the pig fed the basal diet and orally infused with the procine rotavirus; (D) the pig fed the LGG supplementing diet and orally infused with the procine rotavirus. (Original magnification, 100 ×).
(TIF)
