# Supplementary figures and images for: Dietary Lactobacillus rhamnosus GG Supplementation Improves the Mucosal Barrier Function in the Intestine of Weaned Piglets Challenged by Porcine Rotavirus
Source: PLoS One. 2016 Jan 4;11(1):e0146312. doi: 10.1371/journal.pone.0146312 (PMC4699646; doi:10.1371/journal.pone.0146312)

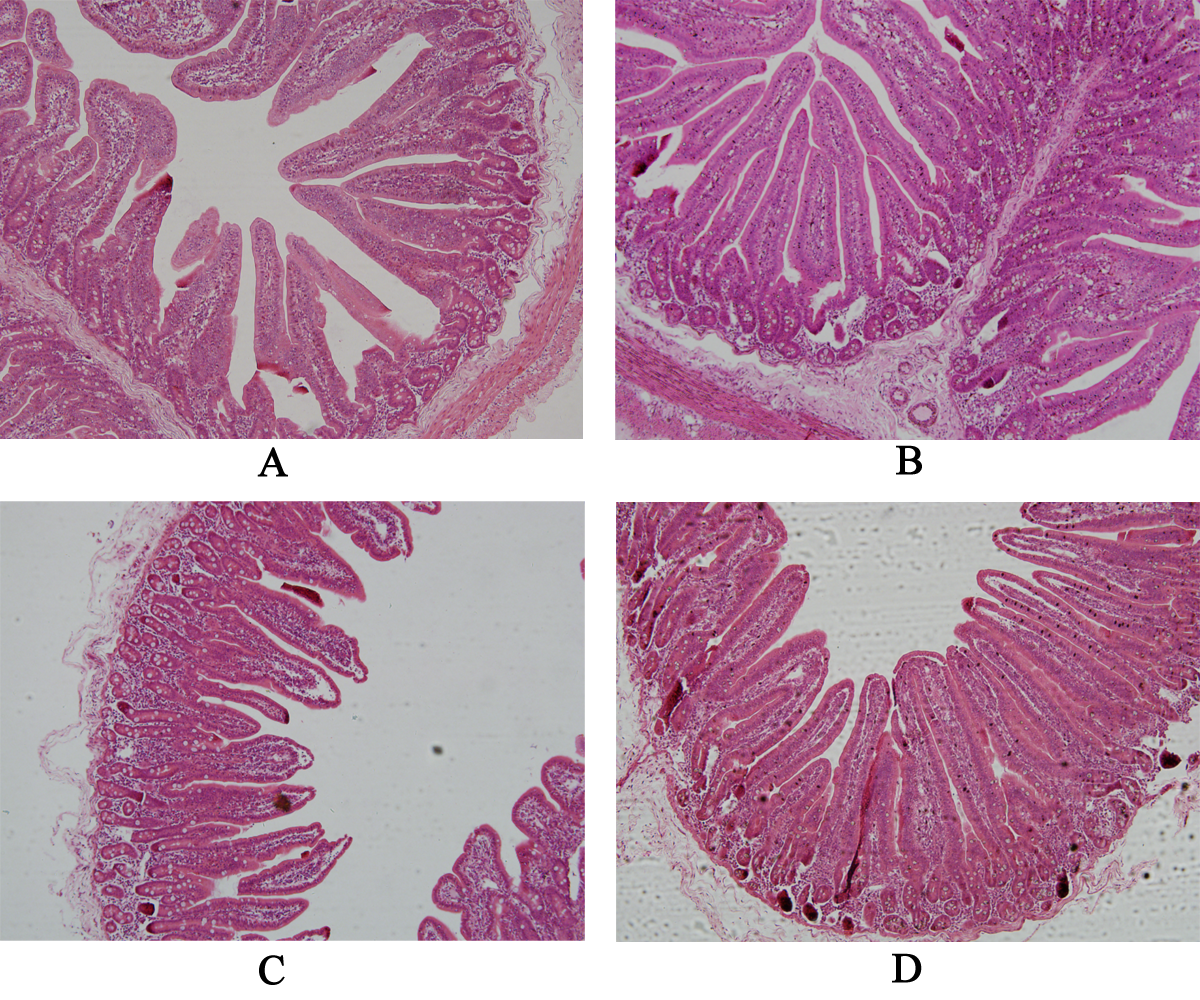

Supplement: S1 Fig — (A) The pig fed the basal diet and orally infused with the sterile essential medium; (B) the pig fed the LGG supplementing diet and orally infused with the sterile essential medium; (C) the pig fed the basal diet and orally infused with the procine rotavirus; (D) the pig fed the LGG supplementing diet and orally infused with the procine rotavirus. (Original magnification, 100 ×). (TIF) [file pone.0146312.s001.tif]
